# The Behaviour Support Plan Content Appraisal Tool (BSP‐CAT): A New Tool for Assessing and Improving the Quality of Behavioural Support Plans

**DOI:** 10.1111/jar.70153

**Published:** 2025-11-24

**Authors:** Peter Baker, Nick Gore, Suzi Sapiets, Jill Chaplin, Mark Murphy, Ceridwen Evans

**Affiliations:** ^1^ Tizard Centre, Cornwallis North East University of Kent Canterbury UK; ^2^ School of Social Policy and Society Intellectual Disabilities Research Institute (IDRIS), University of Birmingham Birmingham UK; ^3^ Northumberland Community Learning Disability Team, Cumbria, Northumberland Tyne and Wear NHS Foundation Trust Blyth UK; ^4^ Independent Chartered Clinical Psychologist

**Keywords:** behaviour support plans, behaviours that challenge, intellectual disability, reliability/audit

## Abstract

**Background:**

People with intellectual disabilities are at increased risk of presenting behaviours that challenge. Behaviour support plans (BSPs) guide the use of bespoke strategies to maximise life quality and reduce the risk of behaviours that challenge. The behaviour support plan content appraisal tool (BSP‐CAT) was developed to assess and improve the quality of BSPs. The aim of this study was to examine the reliability of the BSP‐CAT.

**Method:**

Test–retest and inter‐rater reliability were examined with the assistance of practitioners that supported adults with intellectual disabilities and behaviour that challenges. All participants received prior training and independently used the measure to score BSPs available to them.

**Results:**

Participants provided 98 BSP‐CAT scores for 47 behaviour support plans. Good to moderate reliability was established at the total and strand level.

**Conclusions:**

Preliminary analysis suggests the BSP‐CAT has encouraging reliability.

**Summary**

- People with intellectual disabilities are often at risk of presenting behaviours that challenge.
- Behaviour Support Plans are used to develop and coordinate personalised strategies that improve quality of life and reduce the risk of such behaviours.
- We created the Behaviour Support Plan Content Appraisal Tool (BSP‐CAT) to evaluate and improve the quality of these plans.
- This study aimed to assess how reliable the BSP‐CAT is.
- Early results show that the BSP‐CAT is a potentially reliable tool for developing and assessing the quality of Behaviour Support Plans.

## 1 Introduction

People with intellectual disabilities are at increased risk of displaying behaviours that challenge, such as self‐injury, aggression and property damage (e.g., Bailey et al. 2019; Bowring et al. 2017, 2019; Gore et al. 2013). Behaviours that challenge increase the likelihood of inequalities for people with intellectual disabilities, including heightened risk of abuse and restrictive practices, such as restraint, seclusion, and over‐medication (Allen et al. 2009; Bowring et al. 2017; Male 2003). In addition, people with intellectual disabilities who display behaviours that challenge and their families are at heightened risk of isolation, separation, trauma, stress, and mental health conditions (e.g., Bowring et al. 2019; Gore et al. 2022).

Many people with intellectual disabilities require additional support from others (family carers, paid carers, support workers, etc.), meaning their care is mediated through a range of other people. It is vital that the efforts of these carers are consistent and coordinated to deliver high‐quality care. Behaviour support plans (BSPs) are a key component in ensuring high‐quality care. BSPs are documents that set out a coherent range of strategies to be used when supporting an individual whose behaviour challenges (McVilly et al. 2013; O'Neill et al. 1997). The importance of BSPs is highlighted in guidance by the National Institute for Health and Care Excellence (NICE) on intellectual disabilities and behaviour that challenges (National Institute for Health and Care Excellence (NICE) 2015, 2018) and is recognised as an integral aspect of Positive Behavioural Support (PBS) (Simonsen et al. 2010; Gore et al. 2022). Furthermore, evidence suggests that when BSPs are not used, there are risks of ineffective, inefficient, and harmful interventions being prescribed and implemented (Steege and Watson 2009; Sugai et al. 1999).

To ensure quality, several BSP audit tools have been developed (Benazzi et al. 2006; Browning‐Wright et al. 2013; Horner et al. 2000, 2004; Kroeger et al. 2007; Phillips et al. 2010; Quigley et al. 2018; Tarbox et al. 2013; Van Acker et al. 2005; Vassos et al. 2023; Vollmer et al. 1992; Williams and Vollmer 2015). In a comprehensive review of the use of BSPs in the PBS literature, Vassos et al. (2023) found the BIP‐QEII (Behaviour Intervention Plan Quality Evaluation Version 2), formerly the Behaviour Support Plan Quality Evaluation Guide II (BSP‐QEII) (Browning‐Wright et al. 2013), to be the most frequently utilized tool for BSP quality assessment in PBS studies.

The BIP‐QEII was developed in California in response to legislation mandating that schools conduct functional behaviour assessments and develop behaviour support plans for children within special education who are at risk of engaging in behaviours that challenge (Individuals With Disabilities Education Act 1997; Individuals With Disabilities Education Improvement Act 2004). Legal challenges to the adequacy and validity of existing functional behaviour assessments and behaviour support plans, as well as growing research evidencing best practice for behaviour support plans, led to the development of the initial version of the BIP‐QEII (Browning‐Wright et al. 2007, 2013).^1^

The BIP‐QEII has good established reliability and has been found to be easy and quick to use, providing objective standards for auditing, training and feedback (Browning‐Wright et al. 2007, 2013; Cook et al. 2007; McVilly et al. 2013; Webber et al. 2011). However, the applicability to adults and to populations outside of the US is questionable. In a systematic exploratory analysis of studies employing the BIP‐QEII, Steeples (2022) identified 16 relevant studies. Of these, six of the seven studies that received the lowest quality rating ‘Weak’ focused on adult populations outside the United States (Bosco et al. 2019; Hassiotis et al. 2018; McVilly et al. 2013; O'Dwyer et al. 2017; Wardale et al. 2018; Webber et al. 2011). The remaining study rated as ‘Weak’ involved a mixed sample consisting of approximately 70% adult and 30% child behaviour intervention plans; notably, the children's plans were derived from care settings rather than educational contexts (Webber et al. 2011).

This raises questions regarding the sensitivity of the BIP‐QEII for both adults and for populations outside of the US, specifically the possibility of a floor effect. Chaplin et al. (2014) identified one possible cause for this, noting concerns about the interrelated nature of the components scored, where information from one item informs subsequent assessed areas and interventions. Specifically, this related to the requirement for a Functionally Equivalent Replacement Behaviour (FERB) to be taught. Whilst the importance of supporting the development of an individual's skills is acknowledged and for many individuals the inclusion of a FERB in their BSP would be invaluable, the necessity of a FERB may not be universally applicable. Empirical evidence indicates that the efficacy of functional communication training (FCT)—which entails the teaching of FERBs—may be compromised in contexts where the behaviour that challenges continue to be intermittently or consistently reinforced. This would be the case with automatically reinforced behaviour and suggests that teaching a FERB may not always be sufficient (Shirley et al. 1997). Furthermore, the requirement to teach a FERB for automatically reinforced behaviours is not universally supported in behaviour analysis literature, where best practice is often considered to involve primarily ensuring access to appropriate sensory experiences within the context of a multi‐component plan, that is likely also to include other areas of skill development (Carr and Durand 1985; Cooper et al. 2007).

There is also some evidence that the teaching of a FERB maybe less relevant for adults with intellectual disabilities compared to children (McClean et al. 2005). Research suggests that interventions for adults often place greater emphasis on environmental modifications and proactive support strategies than on structured FERB training (Vassos et al. 2023). These findings highlight the importance of adapting behaviour support approaches to the specific needs and contexts of each population. For this reason, in developing the BSP‐CAT, we concluded that while the teaching of a FERB is often valuable and in many cases essential, it should not be considered an absolute requirement in all circumstances.

Some other technical aspects of the BIP‐QEII—such as the requirement for the BSP to be based on a functional behaviour assessment and the need to address the environmental context that evokes the behaviour—remain highly relevant outside of the United States. However, we considered several key elements regarded as essential to PBS in the United Kingdom (Gore et al. 2013, 2022) to be either inadequately addressed or absent from the BIP‐QEII. These include aligning BSPs with person‐centred goals and aspirations, considering relationships with friends, family, and the wider community, promoting collaborative partnerships, and ensuring that BSPs are practical and feasible by accounting for contextual factors and the needs of those responsible for implementation. McVilly et al. (2013) made similar recommendations and suggested that BSPs should also include key background details of the person reflecting current nomenclature and service philosophy and address contemporary issues such as stakeholder involvement and systems change. These observations prompted work to develop a new tool focused on behaviour support planning for adults with intellectual disabilities in the UK.

A working party of practice‐focused researchers from the [details omitted for peer review], led by the second and first authors created the behaviour support plan—content appraisal tool (BSP‐CAT) over a period of 5 years. The tool was designed to help assess the quality of behaviour support plans (BSPs) created to support adults with intellectual disabilities in the context of behaviours that challenge in UK community settings. The first iteration of the tool was grounded in a review of relevant literature and with close reference to the biopsychosocial model of behaviour that challenges (Hastings et al. 2013), the UK definition of PBS (Gore et al. 2013), and associated PBS resources developed in the UK by the PBS academy (PBS Academy 2015). The development of the tool involved multiple rounds of writing, workshop discussions with stakeholders, and feedback from researchers, practitioners, and family members, alongside incorporation of the refreshed definition of PBS in the UK (Gore et al. 2022).

The BSP‐CAT was specifically developed to evaluate comprehensive, individualised BSPs for individuals whose behaviours that challenge, due to their nature and severity, necessitate a full functional behavioural assessment, alongside person‐centred approaches and other relevant evaluations. These plans, necessitated by the intensity, frequency, duration and impact of the behaviour(s) that challenge, would be comprehensive, largely proactive, multi‐component and include crisis management as a component.

Comprehensive assessment for individuals with intellectual disabilities and behaviours that challenge should consider developmental history, physical and mental health, social and environmental context, the quality of current support, and the capacity of caregivers (National Institute for Health and Care Excellence (NICE) 2015). As everyday support must be informed by these factors, any meaningful audit of a BSP should seek clear evidence that the strategies it contains are both individualised and grounded in such assessments. Furthermore, due to the complex and multifaceted nature of support required and variation employed in the format of BSPs, key behaviour support planning strategies may also be documented outside the BSP itself. Therefore, to ensure a comprehensive representation of the support provided and to confirm that the strategies are clearly derived from comprehensive assessment, we concluded that any audit should also include relevant supplementary documentation—such as functional behaviour assessments, person‐centred plans, health action plans, communication passports and hospital passports.

Examining reliability is essential when evaluating a new measure to ensure its consistency and dependability. Assessing the consistency of results across different raters or observers (inter‐rater reliability) is critical to minimize subjectivity and enhance generalizability. Evaluation of the measure's stability over time (test–retest reliability) by comparing results from repeated administrations further ensures consistency over time and provides evidence for its suitability for long‐term use. Accordingly, the present study aimed to examine the inter‐rater reliability and test–retest reliability of the BSP‐CAT, thereby enhancing confidence in its use for research and practice.

## 2 Methods

### 2.1 Materials

#### 2.1.1 BSP‐Cat

The BSP‐CAT version^2^ as evaluated in the current study was made available to participants in digital format and comprised 15 components relating to aspects of a BSP, organised into six strands (see Table 1). Strand A, Foundations for Support comprised three components reflecting information regarding assessment of the person and their environment to personalise interventions, supports and strategies. Strand B, Enabling Environments comprised seven components reflecting interventions and supports aimed at creating an environment for the person designed to enhance quality of life and create positive life opportunities. Strand C, Antecedent Interventions consisted of two components: proactive interventions to reduce the likelihood of behaviours that challenge occurring and secondary interventions targeting early warning signs to manage immediate situations safely. Strand D, Skills Development comprised one component addressing interventions and supports aimed to increase the person's adaptive behavioural repertoire. Strand E, Reactive Strategies comprised one component outlining responses to incidents of behaviours that challenge when secondary interventions have not been successful, ensuring the safety of all involved. Lastly, Strand F, Implementation and Monitoring of the plan comprised one component detailing guidance on how and by whom interventions, supports and strategies are to be delivered, monitored and assessed, and how the BSP will be modified accordingly. Each component of the BSP‐CAT included a description, rationale and scoring examples.

**TABLE 1:** Strands and components in the BSP‐CAT.

Strands	Components
A. Foundations for support	A1. Formulation
	A2. Contextual fit
	A3. Goals/aspirations
B. Enabling environments	B1. Communication
	B2. Choice
	B3. Physical health
	B4. Mental health
	B5. Relationships with friends, family and the wider community
	B6. Safe, consistent and predictable environment
	B7. High levels of participation in meaningful activity
C. Antecedent interventions	C1. Antecedent interventions
	C2. Secondary prevention interventions
D. Skills development	D1. Skills development
E. Reactive strategies	E1. Reactive strategies
F. Implementation and monitoring of the plan	F1. Monitoring and evaluation of support

Each BSP‐CAT component was scored on a four‐point scale (0–3) based on the quantity and quality of supporting evidence in the BSP and other relevant documentation. Scoring criteria were specified for each component with illustrative examples provided. Higher scores indicate stronger evidence that the component has been adequately addressed in the BSP and/or other relevant documentation.

#### 2.1.2 Data Collection Form

For this study, we developed a data collection form to enable anonymous sharing of BSP‐CAT scores. In addition, the date the participant scored the BSP and codes for linking the participant, service and the BSP were also collected on the form. No further information was collected regarding the individuals for whom the BSPs were created.

#### 2.1.3 Data Collection Instructions

Detailed instructions were provided on the selection of the BSP and the number and timings of the data collection. A copy of the data collection instructions and collection forms is available in Table S1 in the Supporting Information.

#### 2.1.4 Participant Questionnaire

A short participant questionnaire was developed to identify the organisation the participant worked for, the type of service (e.g., education, health, social care, community support), any training or formal qualifications they had received relevant to PBS and/or behaviour support planning, and the number of years they had worked using a PBS framework.

### 2.2 Procedure

The University of (details omitted for peer review) Research Ethics Committee granted ethical approval for the study (reference 568). Practitioners who worked in a UK service supporting at least one adult (18+ years of age) with an intellectual disability and behaviour that challenges where a functional assessment had taken place and a BSP available for review were eligible to take part in the study.

To recruit participants, we shared information about the study with members of professional networks and contacts who had previously expressed interest in the BSP‐CAT. All who expressed interest were sent an information sheet and consent form. Twenty‐nine practitioners who worked in ten organisations/services supporting adults with intellectual disabilities and behaviour that challenges took part in the study. The type of services participants worked for included social care, supported living, community support, education, an integrated health and social care service, a specialist behaviour support team within a local authority, a charity providing services, and a consultancy service for health, social and education service providers. Participants' experience working within a PBS framework ranged from 6 months to 22 years.

Participants' training or formal qualifications relevant to PBS or behaviour support planning varied. Eighteen (62%) had postgraduate/UK Society for Behaviour Analysis (UKSBA) registration/Board Certified Behaviour Analyst (BCBA) level qualification, 2 (7%) had an undergraduate qualification, 2 (7%) had a business and technology education council (BTEC) qualification, 6 participants (21%) had what was described as basic PBS training and one person had no specific PBS training, although they were a qualified learning disability nurse.

Those who returned the consent form were invited to attend a half day (3 h) information/induction session in use of the BSP‐CAT. The session was delivered remotely via Microsoft Teams on two separate dates in July 2022. The session covered: (1) Background and development of the BSP‐CAT; (2) Structure of the tool; (3) Demonstration of the tool and scoring; (4) A scoring exercise (each participant scored a fictional BSP using the BSP‐CAT); (5) Feedback and group discussion on the scoring exercise. Following the session, participants were asked to independently assess a further fictional BSP which had been previously rated by the research team using the BSP‐CAT. After completing their ratings participants received individualized feedback via email, detailing the degree of alignment or discrepancy between their scores and those of the research team, as well as an explanation of the rationale underpinning the research team's evaluations.

#### 2.2.1 Data Collection

The research team sent participants the questionnaire concerning demographics and detailed standardized instructions on how to collect and share BSP‐CAT data with the research team via email. Organizations with one participant were asked to collect test‐retest data, and organizations with two or more participants were asked to collect both inter‐rater and test‐retest data. To collect test–retest data, participants were asked to use the BSP‐CAT to score the same BSP on two separate occasions with a 2–3‐week interval. To gather inter‐rater data, two participants from the same service/organization independently assessed the same BSP documents using the BSP‐CAT on the same occasion. Participants completed the data collection form for each set of BSP documents they scored (BSP‐CAT component scores, date scored, participant code, service code and BSP code). Completed questionnaires and data collection forms were shared with the research team via encrypted email. Participants were informed that this was an opportunistic sample and that, to capture the full range of BSP quality, all BSPs available to them would be considered eligible for review regardless of perceived quality.

#### 2.2.2 Analysis

Data were inputted into Microsoft Excel and IBM SPSS (version 28) software for analysis. Descriptive statistics (mean, standard deviation, range) were calculated for the BSP‐CAT total.

Reliability was assessed at the level of total and strands and with the exception of strands D, E and F (each of which comprised only a single component), strand scores were calculated by summating component scores.

For test–retest data, Pearson's correlation and Spearman's rho (for skewed data) were conducted to examine time 1 and time 2 scores of BSPs for the overall BSP‐CAT total and the strand totals. The interval between the two time points when participants scored the BSP varied from 14 to 111 days (*M* = 46.7, SD = 25.6). Test–retest data with an interval of greater than 60 days between participant ratings were excluded from analyses. As a result 3 plans were excluded. Subsequently, data on 16 BSPs were included in test–retest analyses, with an interval of 14–56 days (*M* = 37.8 days, SD = 15.6) between ratings. Each participant scored between 1 and 10 plans (mean = 7, SD = 4.05).

De Ridder and Wouters (2021) classified Spearman's *ρ* values of 0.40–0.69 as moderate test–retest reliability, 0.70–0.89 as strong, and 0.90–1.00 as very strong. According to Cohen (1988), Pearson's *r* values of 0.10–0.29 indicate a small effect size, 0.30–0.49 a medium effect, and 0.50 or higher a large effect.

For inter‐rater data intra‐class correlations (ICC) were conducted to examine rater 1 and rater 2 scores of the BSP‐CAT total and the strand totals. A total of 35 BPSs were evaluated, resulting in 70 BSP‐CAT scores. Of these, 31 plans were used exclusively for the analysis of inter‐rater reliability, with 4 of those also included in the test–retest assessment. ICC estimates and their 95% confidence intervals were calculated in SPSS based on an average measure one‐way random effects model. Interpretation of ICC values followed the guidelines proposed by Koo and Li (2016), where values less than 0.50 indicate poor reliability, 0.50–0.75 indicate moderate reliability, 0.75–0.90 indicate good reliability, and greater than 0.90 indicate excellent reliability.

#### 2.2.3 Feedback Follow up

Following data analysis, we invited participants to attend a 1‐h follow‐up workshop, delivered remotely via Microsoft Teams on two separate dates in September 2023. Fifteen professionals attended a follow‐up workshop. In the workshops, researchers shared a summary of the findings and collected additional feedback from participants on the BSP‐CAT. This included discussions on ways to improve specific components that were identified as needing greater clarity based on participant feedback and reliability statistics. Qualitative feedback from participants on the BSP‐CAT was also collected during the study via emails participants sent to the research team.

## 3 Results

Participants provided 98 BSP‐CAT scores for 47 BSPs, including test–retest data for 16 BSPs and inter‐rater data for 35 BSPs. Four BSPs had overlapping inter‐rater and test–retest data, where one participant scored the BSP twice and another participant scored it once. In these cases, the first scores from the participant who scored the test–retest were used for the inter‐rater analysis. The BSPs were chosen opportunistically, based on the information available to participants at the time and, as such, their quality could not be evaluated prior to inclusion. The mean BSP‐CAT total score for the included plans was 28.9 (range: 8–42, SD: 15.4), indicating a broad spectrum of plan quality and suggesting that the sample was reasonably representative.

### 3.1 Test–Retest Reliability

Table 2 reports the correlation coefficients and interpretative benchmarks between participants' time 1 and time 2 scores of BSPs. There were significant strong positive correlations with strong or large effects for the BSP‐CAT total and Strands A B, and E, and a significant strong positive correlation with moderate effect sizes for Strand D. There were significant moderate positive correlations for Strand C with a moderate effect size.

**TABLE 2:** Correlation coefficients for test–retest data.

BSP‐CAT variable	Correlation coefficient		Benchmark	Sig.	CI	
	Pearson	Spearman's rho			Lower	Upper
Totals						
BSP‐CAT total	0.859**		Large	< 0.001	0.666	1.000
Strand A		0.757**	Strong	< 0.001	0.478	1.000
Strand B	0.913**		Large	< 0.001	1.000	0.785
Strand C		0.453*	Moderate	0.039	0.019	1.000
Strand D		0.671**	Moderate	0.002	0.329	1.000
Strand E		0.768**	Strong	< 0.001	0.497	1.000
Strand F		0.632**	Moderate	0.004	0.268	1.000

*Note:* Sig 1‐tailed. *p = < 0.05, **p < 0.001.

Abbreviation: CI, confidence interval.

### 3.2 Inter‐Rater Reliability

Table 3 reports the average measures, one‐way random effects model ICC for rater 1 and rater 2 scores of BSPs. A good (and significant) degree of reliability was found between participants' scores of BSPs for Strands C, D, E and F. A moderate (and significant) degree of reliability was found between participants' scores of the BSP‐CAT total and Strands A and B.

**TABLE 3:** Intraclass correlation coefficients for inter‐rater data.

BSP‐CAT component/variable	ICC	Benchmark	95% CI		Value	*F* test with true value 0		Sig.
			Lower	Upper		df1	df2	
Totals								
BSP‐CAT total	0.745	Moderate	0.498	0.871	3.920	34	35	< 0.001**
Strand A	0.544	Moderate	0.102	0.769	2.193	34	35	0.012*
Strand B	0.618	Moderate	0.249	0.807	2.619	34	35	0.003*
Strand C	0.879	Good	0.762	0.939	8.281	34	35	< 0.001**
Strand D	0.775	Good	0.558	0.886	4.453	34	35	< 0.001**
Strand E	0.790	Good	0.588	0.894	4.771	34	35	< 0.001**
Strand F	0.827	Good	0.660	0.912	5.783	34	35	< 0.001**

*Note:* Average measures, one‐way random effects model. *p < 0.05, **p < 0.01 level.

Abbreviations: CI, confidence interval; ICC, intraclass correlation.

### 3.3 Qualitative Feedback

Based on this analysis and the qualitative feedback from participants minor refinements were made to develop the final version of the BSP‐CAT. In particular, the scoring system was reviewed to ensure greater logical consistency throughout (see Table 4). This review was guided by the principle that an effective audit should assess both the presence of a clear statement linking evidence to prior assessment and person‐centred processes and the degree to which the scoring requirements for each component were sufficiently met.

**TABLE 4:** BSP‐CAT scoring final version.

Scoring	
**3**	A score of **3** should be given if there is evidence the component has adequately addressed **BOTH** of the following: There is a clear statement and linked evidence to prior assessment. **AND** All aspects of the scoring requirements for the component are adequately addressed, properly defined, specific, and with appropriate scope and detail.
**2**	A score of **2** should be given if **EITHER** of the following apply: There is unclear or missing statements and linked evidence to prior assessment. **OR** Important aspects of the scoring requirements for the component are missing, inadequate, poorly defined, nonspecific or limited in scope and detail.
**1**	A score of **1** should be given if **BOTH** of the following apply: There is unclear or missing statements and linked evidence to prior assessment. **AND** Important aspects of the scoring requirements for the component are missing, inadequate, poorly defined, nonspecific or limited in scope and detail.
**0**	A score of **0** should be given there is **NO** evidence of any consideration of the scoring requirements for the component.

Clarification as to the function of secondary prevention was provided. This was defined as an intervention targeting the initial stages of behavioural escalation, focused on the identification and management of early indicators of distress or agitation to prevent crises, reduce harm, and limit the use of restrictive practice (Harris et al. 1996; Allen 2002). Secondary intervention as a component was relocated from the antecedent strategies section to the reactive strategies section. The antecedent section was thus refined to focus more clearly on stable, long‐term environmental modifications (Gore et al. 2013). An additional amendment, informed by stakeholder feedback, involved distinguishing between strategies designed to promote general mental well‐being and those intended to offer targeted support for individuals with diagnosed mental health conditions. As a result, physical and mental health considerations were merged into a single component, with distinct examples provided to address both areas appropriately.

Participants also provided feedback on tools and resources that could be developed to support the use of the BSP‐CAT (e.g., a manual) and suggested that it would be helpful to explore the use of the BSP‐CAT for other populations (e.g., children with intellectual disabilities).

## 4 Discussion

The BSP‐CAT was developed to assess the content and quality of BSPs designed for adults with intellectual disabilities, particularly in the context of behaviours that challenge in UK community settings. Preliminary analysis of its psychometric properties indicated encouraging inter‐rater and test–retest reliability, both at the total score and strand levels. The tool's development involved broad and comprehensive consultation—including multiple drafting stages, stakeholder workshops, and feedback from researchers, practitioners, and family members—which contributes to its face and content validity. However, validity was not formally assessed in this study and remains an important focus for future research.

Most of the participants in this study had expertise and experience in PBS which likely enhanced the rigour and accuracy of their evaluations of BSPs. This is in keeping with the conclusions derived from the study by Vassos et al. (2023), who recommended that BSP quality auditing tools based on technical compliance with behavioural principles are only utilised by stakeholders with extensive experience/knowledge of PBS.

Whilst the BSP‐CAT has been designed primarily as a tool for appraising the content of BSPs with the aim of directly enhancing the quality of support provided to individuals, it is also hoped that it may serve as a benchmark and guide to support and train those responsible for creating BSPs as a supervision/self‐audit or team practice development tool. The BSP‐CAT may additionally have applications within research to help further explore the evidence base for PBS. Being able to rate the quality of BSPs may allow researchers to determine the extent to which key components of PBS have been implemented in intervention studies (i.e., to determine fidelity of PBS interventions and/or as an outcome for service development interventions). It also will allow researchers to explore how particular outcomes relevant to PBS vary in accordance with BSP quality (both in terms of the overall quality of BSP and the quality of strands of the BSP‐CAT). The continued real‐world use of the BSP‐CAT represents ongoing testing of its face and content validity, framing validity as a continuous process rather than a one‐time assessment. While formal validation remains to be tested, the tool's conceptual coherence and the encouraging psychometric properties demonstrated to date support its utility. As such, the BSP‐CAT provides a viable option for researchers in the field of Positive Behaviour Support (PBS), and its use is both welcomed and encouraged.

### 4.1 Cautions, Caveats and Limitations

All participants in this study were specifically selected as having prior experience and background knowledge in PBS. Therefore, in common with Vassos et al. (2023), we strongly recommend that the BSP‐CAT be used only by individuals with appropriate professional experience and training in PBS. Additionally, whilst the psychometric properties of the BSP‐CAT were broadly encouraging, the need for minor amendments to the version tested will require that the finalised version of the BSP‐CAT will need further specific evaluation. Furthermore, to more robustly establish the generalisability of the measures, additional reliability testing is warranted, employing a consistent cohort of participants to evaluate multiple plans representing a range of quality levels. We would also advise that when used in research any data generated will require additional and specific investigation of reliability.

The amended BSP‐CAT and associated manual are freely available under Creative Commons Attribution Non‐Commercial No Derivatives 4.0 International (CC BY‐NC‐ND 4.0). They may be shared provided the licence terms are followed, including appropriate attribution, non‐commercial use, and no creation of derivative works or additional works. For more information and to download the BSP‐CAT and User's Manual click here (https://pbs‐academy.com/behaviour‐support‐plan‐content‐appraisal‐tool‐bsp‐cat/).

## Author Contributions

Peter Baker Nick Gore (leads), Suzi Sapiets (supporting): conceptualisation. Peter Baker, Nick Gore: methodology. Nick Gore, Suzi Sapiets (leads), Jill Chaplin, Peter Baker (supporting): investigation. Suzi Sapiets (lead) Peter Baker (supporting): formal analysis. Peter Baker (lead): writing – original draft. Peter Baker, Nick Gore, Suzi Sapiets, Jill Chaplin, Mark Murphy: writing – review and editing. Nick Gore. Peter Baker: supervision.

## Funding

The third author was funded by SF‐DDARIN.

## Ethics Statement

The University of Kent's Tizard Centre Research Ethics Committee granted ethical approval for the study (reference 568).

## Conflicts of Interest

The authors declare no conflicts of interest.

## Supporting information


**Data S1:** Supporting Information.

## Data Availability

The data that support the findings of this study are available on request from the corresponding author. The data are not publicly available due to privacy or ethical restrictions.

## Endnotes

^1^
BIPQEII is no longer available on the PENT website, and a new measure called the Essential 10 has taken its place.

^2^
The finalised version of the BSP‐CAT contains refinements made based on the results of this study.

## References

[jar70153-bib-0001] Allen, D. 2002. “Devising Individualised Risk Management Plans.” In Ethical Approaches to Physical Intervention: Responding to Challenging Behaviour in People With Severe Intellectual Disabilities, edited by D. Allen , 71–88. BILD.

[jar70153-bib-0002] Allen, D. , K. Lowe , P. Baker , et al. 2009. “Assessing the Effectiveness of Positive Behavioural Support: The CPO Project.” International Journal of Positive Behavioural Support 1, no. 1: 14–23.

[jar70153-bib-0003] Bailey, T. , V. Totsika , R. P. Hastings , C. Hatton , and E. Emerson . 2019. “Developmental Trajectories of Behaviour Problems and Prosocial Behaviours of Children With Intellectual Disabilities in a Population‐Based Cohort.” Journal of Child Psychology and Psychiatry 60, no. 11: 1210–1218. 10.1111/jcpp.13080. 31225660

[jar70153-bib-0004] Benazzi, L. , R. H. Horner , and R. H. Good . 2006. “Effects of Behavior Support Team Composition on the Technical Adequacy and Contextual Fit of Behavior Support Plans.” Journal of Special Education 40, no. 3: 160–170. 10.1177/00224669060400030401.

[jar70153-bib-0005] Bosco, A. , L. Paulauskaite , I. Hall , et al. 2019. “Process Evaluation of a Randomised Controlled Trial of PBS‐Based Staff Training for Challenging Behaviour in Adults With Intellectual Disability.” PLoS One 14, no. 8: e0221507. 10.1371/journal.pone.0221507. 31437228 PMC6705827

[jar70153-bib-0006] Bowring, D. L. , J. Painter , and R. P. Hastings . 2019. “Prevalence of Challenging Behaviour in Adults With Intellectual Disabilities, Correlates, and Association With Mental Health.” Current Developmental Disorders Reports 6, no. 4: 173–181. 10.1007/s40474-019-00175-9.

[jar70153-bib-0007] Bowring, D. L. , V. Totsika , R. P. Hastings , S. Toogood , and G. M. Griffith . 2017. “Challenging Behaviours in Adults With an Intellectual Disability: A Total Population Study and Exploration of Risk Indices.” British Journal of Clinical Psychology 56, no. 1: 16–32. 10.1111/bjc.12118. 27878840

[jar70153-bib-0008] Browning‐Wright, D. , G. R. Mayer , C. R. Cook , S. D. Crews , B. R. Kraemer , and B. Gale . 2007. “A Preliminary Study on the Effects of Training Using Behavior Support Plan Quality Evaluation Guide (BSP‐QE) to Improve Positive Behavioral Support Plans.” Education and Treatment of Children 30, no. 3: 89–106. 10.1353/etc.2007.0017.

[jar70153-bib-0009] Browning‐Wright, D. , G. R. Mayer , and D. Saren . 2013. “Behaviour Intervention Plan Quality Evaluation Scoring Guide II.” The Positive Environments, Network of Trainers. http://www.pent.ca.gov.

[jar70153-bib-0010] Carr, E. G. , and V. M. Durand . 1985. “Reducing Behavior Problems Through Functional Communication Training.” Journal of Applied Behavior Analysis 18, no. 2: 111–126. 10.1901/jaba.1985.18-11. 2410400 PMC1307999

[jar70153-bib-0011] Chaplin, J. , R. P. Hastings , and S. Noone . 2014. “Improving the Quality of Behavioural Support Plans Through Service Development Initiatives.” International Journal of Positive Behavioural Support 4, no. 2: 14–23.

[jar70153-bib-0012] Cohen, J. 1988. Statistical Power Analysis for the Behavioral Sciences. 2nd ed. Routledge.

[jar70153-bib-0013] Cook, C. R. , S. Dean Crews , D. Browning Wright , et al. 2007. “Establishing and Evaluating the Substantive Adequacy of Positive Behavioral Support Plans.” Journal of Behavioral Education 16, no. 3: 191–206. 10.1007/s10864-006-9024-8.

[jar70153-bib-0014] Cooper, J. O. , T. E. Heron , and W. L. Heward . 2007. Applied Behavior Analysis. 2nd ed. Pearson Education.

[jar70153-bib-0015] De Ridder, W. A. , and R. M. Wouters . 2021. “Test–Retest Reliability and Construct Validity of the Satisfaction With Treatment Result Questionnaire in Patients With Hand and Wrist Conditions: A Prospective Study.” Clinical Orthopaedics and Related Research 479, no. 9: 2033–2041. 10.1097/CORR.0000000000001872. 34014631 PMC8373545

[jar70153-bib-0016] Gore, N. J. , P. McGill , S. Toogood , et al. 2013. “Definition and Scope for Positive Behavioural Support.” International Journal of Positive Behavioural Support 3, no. 2: 14–23.

[jar70153-bib-0017] Gore, N. J. , S. J. Sapiets , L. D. Denne , et al. 2022. “Positive Behavioural Support in the UK: A State of the Nation Report.” International Journal of Positive Behavioural Support 12, no. 1: 4–39.

[jar70153-bib-0018] Harris, J. , D. Allen , M. Cornick , A. Jefferson , and R. Mills . 1996. Physical Interventions: A Policy Framework. BILD.

[jar70153-bib-0019] Hassiotis, A. , M. Poppe , A. Strydom , et al. 2018. “Clinical Outcomes of Staff Training in Positive Behaviour Support to Reduce Challenging Behaviour in Adults With Intellectual Disability: Cluster Randomised Controlled Trial.” British Journal of Psychiatry 212, no. 3: 161–168. 10.1192/bjp.2017.34. 29436314

[jar70153-bib-0020] Hastings, R. P. , D. Allen , P. Baker , and S. Toogood . 2013. “A Conceptual Framework for Understanding Why Challenging Behaviours Occur in People With Developmental Disabilities.” International Journal of Positive Behavioural Support 3, no. 2: 6–17.

[jar70153-bib-0021] Horner, R. H. , G. Sugai , A. W. Todd , and T. Lewis‐Palmer . 2000. “Elements of Behavior Support Plans: A Technical Brief.” Exceptionality 8, no. 3: 205–215. 10.1207/S15327035EX0803_6.koo.

[jar70153-bib-0022] Horner, R. H. , A. W. Todd , T. Lewis‐Palmer , L. K. Irvin , G. Sugai , and J. B. Boland . 2004. “The School‐Wide Evaluation Tool (SET): A Research Instrument for Assessing School‐Wide Positive Behavior Support.” Journal of Positive Behavior Interventions 6, no. 1: 3–12. 10.1177/10983007040060010201.

[jar70153-bib-0023] Individuals With Disabilities Education Act . 1997. “20 U.S.C. § 1400.”

[jar70153-bib-0024] Individuals With Disabilities Education Improvement Act . 2004. “20 U.S.C. § 1400.”

[jar70153-bib-0025] Koo, T. K. , and M. Y. Li . 2016. “A Guideline of Selecting and Reporting Intraclass Correlation Coefficients for Reliability Research.” Journal of Chiropractic Medicine 15, no. 2: 155–163. 10.1016/j.jcm.2016.02.012. 27330520 PMC4913118

[jar70153-bib-0026] Kroeger, S. D. , L. J. Phillips , M. Beach , and S. Carolina . 2007. “Positive Behavior Support Assessment Guide: Creating Student‐Centered Behavior Plans.” Assessment for Effective Intervention 32, no. 2: 100–112. 10.1177/15345084070320020101.

[jar70153-bib-0027] Male, D. 2003. “Challenging Behaviour: The Perceptions of Teachers of Children and Young People With Severe Learning Disabilities.” Journal of Research in Special Educational Needs 3, no. 3: 162–171. 10.1111/1471-3802.00011.

[jar70153-bib-0028] McClean, B. , I. M. Grey , and M. McCracken . 2005. “An Evaluation of Positive Behavioural Support for People With Very Severe Challenging Behaviours in Community‐Based Settings.” Journal of Intellectual Disabilities 9, no. 3: 195–207. 10.1177/1744629505056305. 17846050

[jar70153-bib-0029] McVilly, K. , L. Webber , G. Sharp , and M. Paris . 2013. “The Content Validity of the Behaviour Support Plan Quality Evaluation Tool (BSP‐QEII) and Its Potential Application in Accommodation and Day‐Support Services for Adults With Intellectual Disability.” Journal of Intellectual Disability Research 57, no. 8: 703–715. 10.1111/j.1365-2788.2012.01602.x. 22845742

[jar70153-bib-0030] National Institute for Health and Care Excellence (NICE) . 2015. “Challenging Behaviour and Learning Disabilities: Prevention and Interventions for People With Learning Disabilities Whose Behaviour Challenges.” National Institute for Health and Care Excellence. https://www.nice.org.uk/guidance/ng11. 26180881

[jar70153-bib-0031] National Institute for Health and Care Excellence (NICE) . 2018. “Learning Disabilities and Behaviour That Challenges: Service Design and Delivery.” National Institute for Health and Care Excellence. www.nice.org.uk/guidance/ng93.

[jar70153-bib-0032] O'Dwyer, C. , K. R. McVilly , and L. Webber . 2017. “The Impact of Positive Behavioural Support Training on Staff and the People They Support.” International Journal of Positive Behavioural Support 7, no. 2: 13–23.

[jar70153-bib-0033] O'Neill, R. E. , R. H. Horner , R. W. Albin , J. R. Sprague , K. Storey , and J. S. Newton . 1997. Functional Assessment and Program Development for Problem Behavior: A Practical Handbook. 2nd ed. Brooks/Cole.

[jar70153-bib-0034] PBS Academy . 2015. “Positive Behavioural Support: A Competence Framework.” PBS Academy. http://pbsacademy.org.uk/pbs‐competence‐framework/.

[jar70153-bib-0035] Phillips, L. , L. Wilson , and E. Wilson . 2010. “Assessing Behaviour Support Plans for People With Intellectual Disability Before and After the Victorian Disability Act 2006.” Journal of Intellectual and Developmental Disability 35, no. 1: 9–13. 10.3109/13668250903499090. 20121661

[jar70153-bib-0036] Quigley, S. P. , R. K. Ross , S. Field , and A. A. Conway . 2018. “Toward an Understanding of the Essential Components of Behavior Analytic Service Plans.” Behavior Analysis in Practice 11: 436–444. 10.1007/s40617-018-0255-7. 30538920 PMC6269397

[jar70153-bib-0037] Shirley, M. J. , B. A. Iwata , S. Kahng , J. L. Mazaleski , and D. C. Lerman . 1997. “Does Functional Communication Training Compete With Ongoing Contingencies of Reinforcement? An Analysis During Response Acquisition and Maintenance.” Journal of Applied Behavior Analysis 30, no. 1: 93–104. 10.1901/jaba.1997.30-93. 9157100 PMC1284039

[jar70153-bib-0038] Simonsen, B. , L. Britton , and D. Young . 2010. “School‐Wide Positive Behavior Support in an Alternative School Setting: A Case Study.” Journal of Positive Behavior Interventions 12, no. 3: 180–191. 10.1177/1098300708330495.

[jar70153-bib-0039] Steege, M. W. , and T. S. Watson . 2009. Conducting School‐Based Functional Behavioral Assessments: A Practitioner's Guide. 2nd ed. Guilford Press.

[jar70153-bib-0040] Steeples, M. 2022. “A Systematic Exploratory Analysis of the ‘Behavior Support Plan Quality Evaluation II’ Tool.” Unpublished Master's Dissertation, Tizard Centre, University of Kent.

[jar70153-bib-0041] Sugai, G. , R. H. Horner , and J. R. Sprague . 1999. “Functional‐Assessment‐Based Behavior Support Planning: Research to Practice to Research.” Behavioral Disorders 24, no. 3: 253–257. 10.1177/019874299902400309.

[jar70153-bib-0042] Tarbox, J. , A. C. Najdowski , R. Bergstrom , et al. 2013. “Randomized Evaluation of a Web‐Based Tool for Designing Function‐Based Behavioral Intervention Plans.” Research in Autism Spectrum Disorders 7, no. 12: 1509–1517. 10.1016/j.rasd.2013.08.005.

[jar70153-bib-0043] Van Acker, R. , L. Boreson , R. A. Gable , and T. Potterton . 2005. “Are We on the Right Course? Lessons Learned About Current FBA/BIP Practices in Schools.” Journal of Behavioral Education 14, no. 1: 35–56. 10.1007/s10864-005-0960-5.

[jar70153-bib-0044] Vassos, M. , T. Carberry , F. Davis , S. Wardale , and K. Nankervis . 2023. “Can the Behaviour Intervention Plan Quality Evaluation, Version 2, Be Simplified for Use by Stakeholders With Limited Experience of Positive Behaviour Support?” Journal of Intellectual Disability Research 67, no. 5: 488–497. 10.1111/jir.13020. 36815279

[jar70153-bib-0045] Vollmer, T. R. , B. A. Iwata , J. R. Zarcone , and T. A. Rodgers . 1992. “A Content Analysis of Written Behavior Management Programs.” Research in Developmental Disabilities 13, no. 5: 429–441. 10.1016/0891-4222(92)90001-M. 1410711

[jar70153-bib-0046] Wardale, S. , F. Davis , M. Vassos , and K. Nankervis . 2018. “The Outcome of a Statewide Audit of the Quality of Positive Behaviour Support Plans.” Journal of Intellectual & Developmental Disability 43, no. 2: 202–212. 10.3109/13668250.2016.1254736.

[jar70153-bib-0047] Webber, L. S. , K. R. McVilly , T. Fester , and J. Chan . 2011. “Factors Influencing Quality of Behaviour Support Plans and the Impact of Plan Quality on Restrictive Intervention Use.” International Journal of Positive Behavioural Support 1, no. 1: 24–31.

[jar70153-bib-0048] Williams, D. E. , and T. R. Vollmer . 2015. “Essential Components of Written Behavior Treatment Plans.” Research in Developmental Disabilities 36: 323–327. 10.1016/j.ridd.2014.10.003. 25462492

