# Supplementary material for: The Behaviour Support Plan Content Appraisal Tool (BSP‐CAT): A New Tool for Assessing and Improving the Quality of Behavioural Support Plans
Source: J Appl Res Intellect Disabil. 2025 Nov 24;38(6):e70153. doi: 10.1111/jar.70153 (PMC12641237; doi:10.1111/jar.70153)
Supplement: Supplementary file 1 — Data S1: Supporting Information. [file JAR-38-e70153-s001.docx]

**Table S1**

**BSP-CAT Data Collection Form & Instructions**

Complete this form with codes and scores for each behaviour support plan (BSP) scored. **Please only report codes when sharing BSP-CAT scores with the research team and do not include any details about the BSP scored to ensure anonymity**. The codes will link scores for the purposes of inter-rater and test-retest reliability. If you are not sure what codes to use, please contact the research team. Once completed, please send the form to XXXXXXXXXX

| **Linking Codes** | |
| --- | --- |
| **Service/organisation** |  |
| **Participant** (the person who scored the BSP) |  |
| **BSP** (the BSP scored) |  |
| **Date BSP scored** |  |
| **BSP-CAT Scores** | |
| **Component** | **Score** (0/1/2/3) |
| **A1** |  |
| **A2** |  |
| **A3** |  |
| **B1** |  |
| **B2** |  |
| **B3** |  |
| **B4** |  |
| **B5** |  |

**Instructions for services with 1 participant**

Hi [PARTICIPANT NAME],

Thank you so much for your support with the BSP-CAT study so far. We are now able to begin the exciting phase of testing!

As there is only you taking part from [SERVICE/ORGANISATION NAME], we would be grateful if you could test the BSP-CAT’s **test-retest reliability** – which is when the same person uses the BSP-CAT to score the same behaviour support plan on 2 different occasions (with a gap of 2-3 weeks).

Please make use of the guidance and scoring examples for each component in the BSP-CAT, rather than personal interpretations of what needs to be included in a plan to be “good PBS”. Also, **all scoring of plans needs to be done independently, so no looking at your previous scores for the plan you are scoring – this is really important to test the reliability and validity of the BSP-CAT** (remember –we’re testing the BSP-CAT, not you!).

Please see below instructions on how to collect and share testing data with the research team.

*Instructions – testing test-retest reliability*

- Complete ‘behaviour support plan codes’ in the *‘Codes for BSP-CAT’* word document to record details of the plans that will be used to test the BSP-CAT (e.g., initials/name of person the plan was developed for, date the plan was created/updated) – do not share this with the research team – this is for your reference only
- The participant will use the BSP-CAT to score a behaviour support plan and record: the plan’s scores for each BSP-CAT component, the service/organisation code, the participant code (the person who scored the BSP), and the behaviour support plan code (the plan scored)
- The same participant will use the BSP-CAT to **independently** score the same plan **2-3 weeks later** (this needs to be the same version of the plan, and no looking at your previous scores!)
- The participant will share their scores with the research team via email to [XXXXXXXX](mailto:S.Sapiets@kent.ac.uk) – either by completing the *‘BSP-CAT data collection form’* word document or taking a screenshot of the BSP-CAT score summary and sending with the service/organisation, participant, and behaviour support plan codes (this can be after each time scoring the plan or after both times scoring the plan)

Codes for services/organisations and participants have been assigned by the research team (attached to this email as a word document titled *‘Codes for BSP-CAT’*)*.* You will be responsible for assigning codes for behaviour support plans, therefore can use this document to record details of the plans (e.g., initials/name of person the plan was developed for, date the plan was created/updated) which cannot be shared with the research team.

Thank you again so much for your continued support with the study. Please let me know if you have any questions or would like to discuss this in a call.

**Instructions for services with 2+ participants**

Hi everyone,

Thank you all so much for your support with the BSP-CAT study so far. We are now able to begin the exciting phase of testing!

As there is more than one person taking part from [SERVICE/ORGANISATION NAME], we would be grateful if you could test the BSP-CAT’s **inter-rater reliability**. This is when 2 people individually use the BSP-CAT to score the same behaviour support plan (this can be at different times but needs to be the same version of the plan). If you also have time to do so, you can test the BSP-CAT’s **test-retest reliability** – which is when one person uses the BSP-CAT to score a behaviour support plan on two different occasions (with a gap of 2-3 weeks).

Please make use of the guidance and scoring examples for each component in the BSP-CAT, rather than personal interpretations of what needs to be included in a plan to be “good PBS”. Also, **all scoring of plans needs to be done independently, so no looking at your previous scores for the plan you are scoring – this is really important to test the reliability and validity of the BSP-CAT** (remember –we’re testing the BSP-CAT, not you!).

Please see below instructions on how to collect and share testing data with the research team.

*Instructions – testing inter-rater reliability*

- Complete ‘behaviour support plan codes’ in the *‘Codes for BSP-CAT’* word document for plans that will be used to test the BSP-CAT (do not share with the research team – this is for your reference only)
- Agree which plans each participant will score (2 for each plan)
- 2 participants will **individually** score the same plan using the BSP-CAT (this can be at different times but needs to be the same version of the plan, and no talking to the other person rating the plan!) and record: the plan’s scores for each BSP-CAT component, the service/organisation code, the participant code (the person who scored the BSP), and the behaviour support plan code (the plan scored)
- Each participant will share their scores with the research team via email to [XXXXXXXXX](mailto:S.Sapiets@kent.ac.uk) – either by completing the *‘BSP-CAT data collection form’* word document or taking a screenshot of the BSP-CAT score summary and sending with the service/organisation, participant, and behaviour support plan codes

*Instructions – testing test-retest reliability*

- Complete ‘behaviour support plan codes’ in the *‘Codes for BSP-CAT’* word document to record details of the plans that will be used to test the BSP-CAT (e.g., initials/name of person the plan was developed for, date the plan was created/updated) – do not share this with the research team – this is for your reference only
- Agree which plans each participant will score (1 for each plan)
- The participant will use the BSP-CAT to score a behaviour support plan and record: the plan’s scores for each BSP-CAT component, the service/organisation code, the participant code (the person who scored the BSP), and the behaviour support plan code (the plan scored)
- The same participant will use the BSP-CAT to **independently** score the same plan **2-3 weeks later** (this needs to be the same version of the plan, and no looking at your previous scores!)
- The participant will share their scores with the research team via email to [XXXXXXXXX](mailto:S.Sapiets@kent.ac.uk) – either by completing the *‘BSP-CAT data collection form’* word document or taking a screenshot of the BSP-CAT score summary and sending with the service/organisation, participant, and behaviour support plan codes (this can be after each time scoring the plan or after both times scoring the plan)

Codes for services/organisations and participants have been assigned by the research team (attached to this email as a word document titled *‘Codes for BSP-CAT’*)*.* You will be responsible for assigning codes for behaviour support plans, therefore can use this document to record details of the plans (e.g., initials/name of person the plan was developed for, date the plan was created/updated) which cannot be shared with the research team.

Thank you all again so much for your continued support with the study. Please let me know if you have any questions or would like to discuss this in a call.
